# Supplementary figures and images for: Identification of SLC35A1 as an essential host factor for the transduction of multi-serotype recombinant adeno-associated virus (AAV) vectors
Source: mBio. 2024 Nov 27;16(1):e03268-24. doi: 10.1128/mbio.03268-24 (PMC11708056; doi:10.1128/mbio.03268-24)

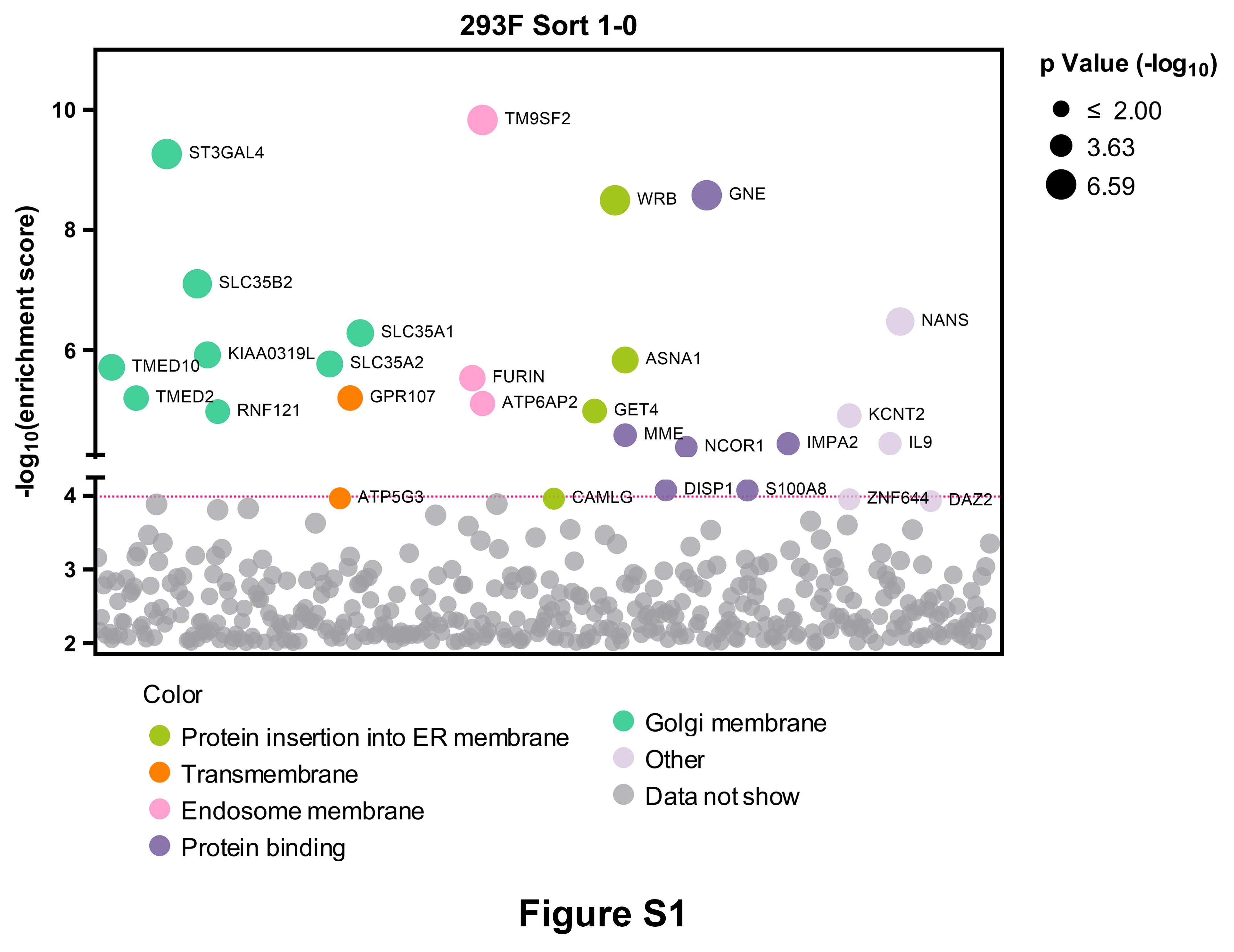

Supplement: Figure S1 — Genes enriched in the first-round screen of mCherry-negative cells. [file mbio.03268-24-s0001.tif]

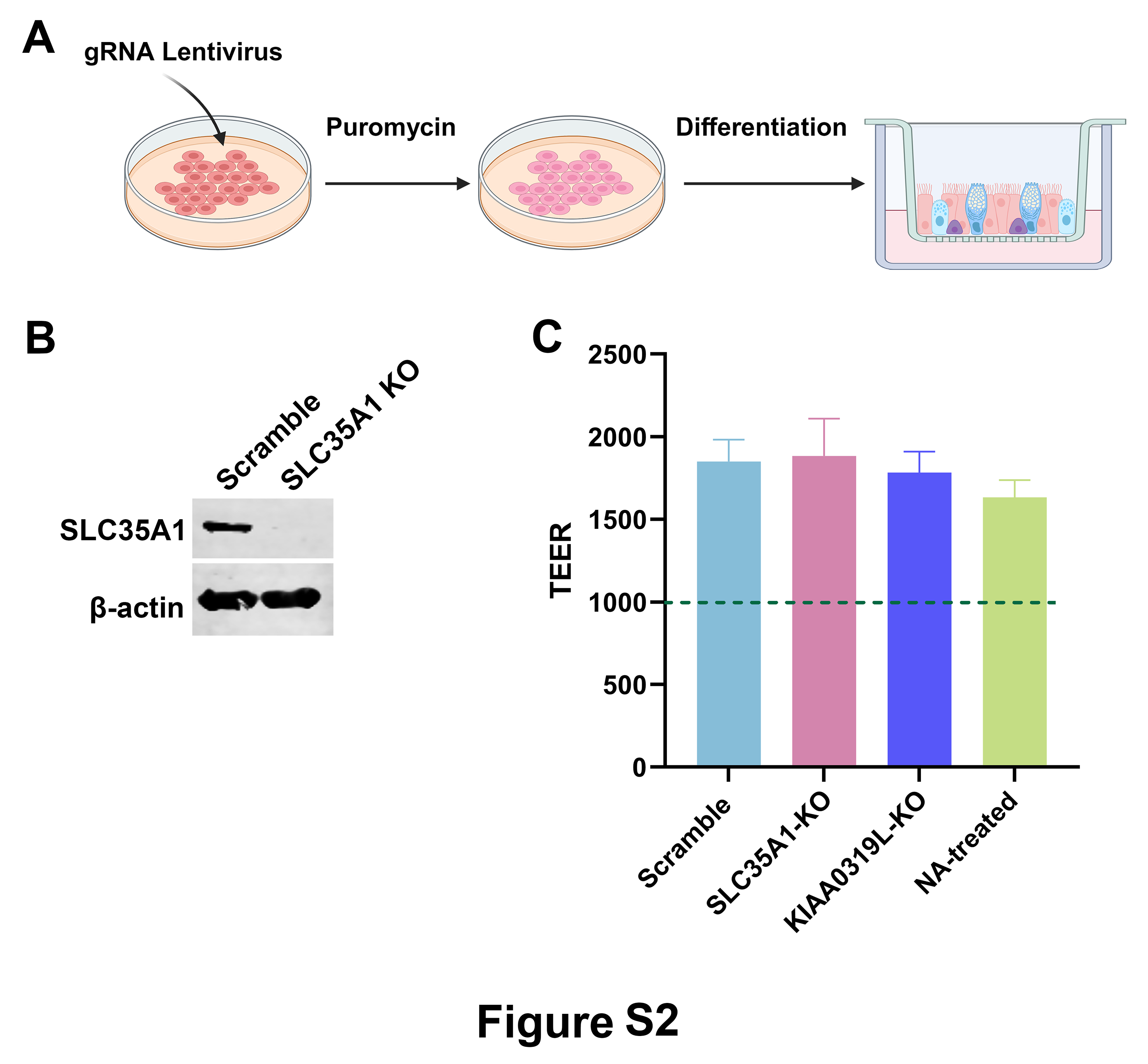

Supplement: Figure S2 — SLC35A1 KO in HAE-ALI culture. [file mbio.03268-24-s0002.tif]

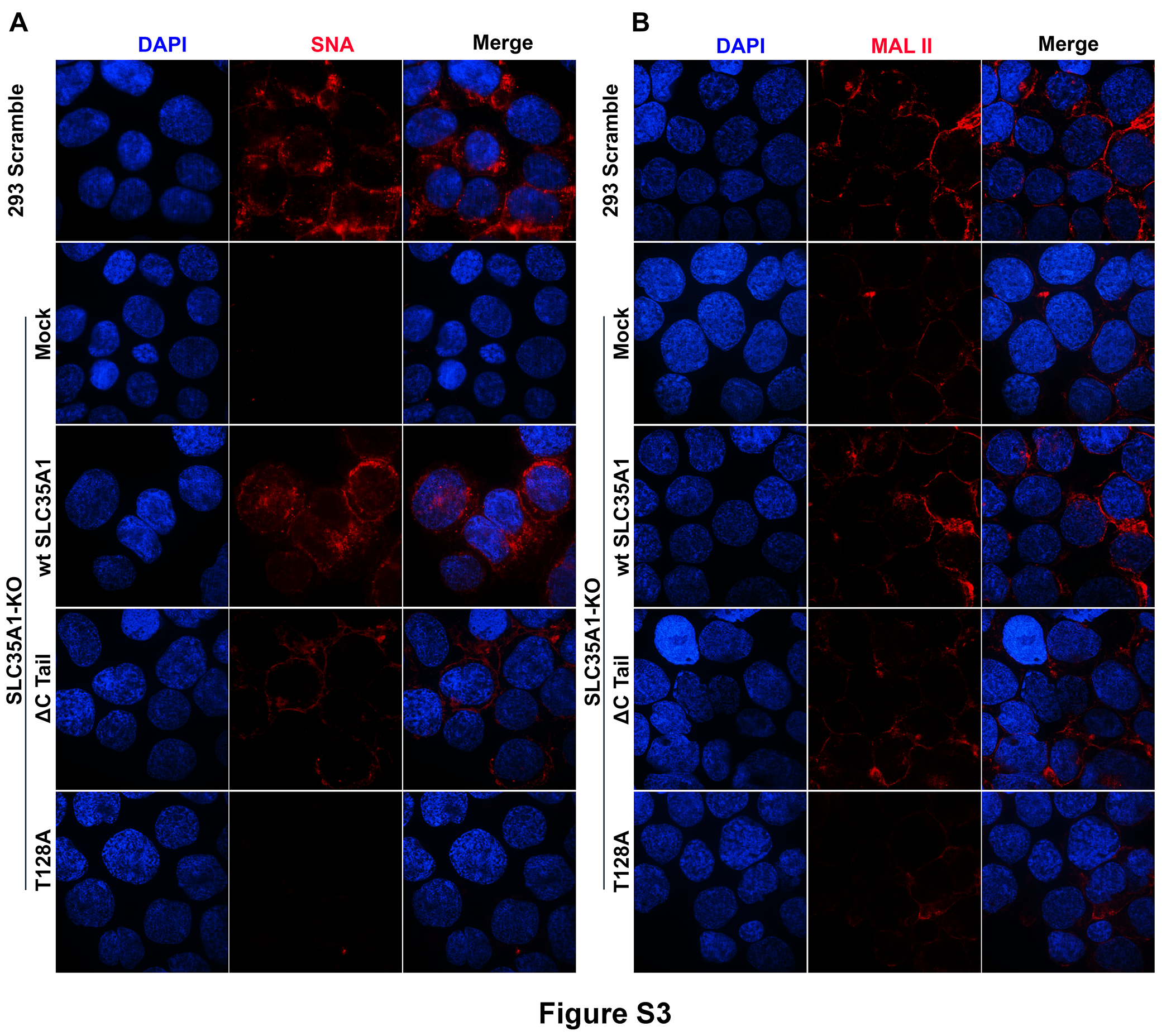

Supplement: Figure S3 — SIA expression. [file mbio.03268-24-s0003.tif]
