# Supplementary material for: Identification of SLC35A1 as an essential host factor for the transduction of multi-serotype recombinant adeno-associated virus (AAV) vectors
Source: mBio. 2024 Nov 27;16(1):e03268-24. doi: 10.1128/mbio.03268-24 (PMC11708056; doi:10.1128/mbio.03268-24)
Supplement: Legends — for supplemental material. [file mbio.03268-24-s0004.docx]

**Supplemental Materials**

**Figure S1. Genes enriched in the first-round screen of mCherry-negative cells.**

The x-axis represents genes targeted by the Brunello library, grouped by GO analysis. The y-axis shows the enrichment score [-log_10_] of each gene based on MAGeCK analysis of the sgRNA reads in gDNA^Sort1^ vs gDNA^sort0^ (**Table S2**). Each circle represents a gene, with its size indicating the statistical significance [-log_10_] of enrichment when comparing gDNA^Sort1^ to gDNA^Sort0^. The color of each circle represents the function of the genes. Only genes with an enrichment score greater than 10^4^ are shown.

**Figure S2. SLC35A1 KO in HAE-ALI culture.**

**(A) Generation of HAE-ALI^SLC35A1-KO^ cultures.** Human airway epithelial cell line CuFi-8 cells were transduced with a gRNA/Cas9 lentivirus. The puromycin resistant cells were seeded onto Transwell inserts and differentiated at an ALI for 4 weeks. **(B) Validation of SLC35A1 expression in HAE-ALI cultures.** Western blotting detected SLC35A1 expression in cultures derived from the scramble control but none in the cultures from *SLC35A1* KO cells. β-actin was detected as a loading control. **(C) Transepithelial electrical resistance (TEER) measurement.** HAE-ALI cultures, Scramble control, SLC35A1-KO, KIAA0319L-KO, and the Scramble control treated with NA were detected for TEER values.

**Figure S3. SIA expression in wide-type or mutant *SLC35A1* expression HEK293^SLC35A1-KO^ cells.**

HEK293^SLC35A1-KO^ cells were mock-treated or transduced with lentiviral vectors expressing SLC35A1 WT, T128A and ∆C Tail mutants, as indicated, followed by selection of blasticidin (at 10 µg/ml) for 2 weeks. The cells were fixed with 4% PFA and then permeabilized with 0.1% Trixon X-100 for intracellular staining. Biotinylated SNA and MAL II lectins were used to stain glycan expression in HEK293^SLC35A1-KO^ cells. SNA (**A**) and MAL II (**B**) stained cells were incubated with DyLight 649-conjugated streptavidin for visualization under a confocal microscope (CSU-W1 SoRa, Nikon) at 60×. HEK293 Scramble cells were used as a control.

**Table S1. A list of genes enriched in the second round (FD400210-FD400208) of the sorted mCherry-negative cells and ranked by the -log_10_ enrichment score.**

**Table S2. A list of genes enriched in the first round (FD400209-FD400208) of the sorted mCherry-negative cells and ranked by the -log_10_ enrichment score.**
